# Detection of Bulbar Involvement in Patients With Amyotrophic Lateral Sclerosis by Machine Learning Voice Analysis: Diagnostic Decision Support Development Study

**DOI:** 10.2196/21331

**Published:** 2021-03-10

**Authors:** Alberto Tena, Francec Claria, Francesc Solsona, Einar Meister, Monica Povedano

**Affiliations:** 1 Information and Communication Technologies Group International Centre for Numerical Methods in Engineering Barcelona Spain; 2 Department of Computer Science Universitat de Lleida Lleida Spain; 3 Institute of Cybernetics Tallinn University of Technology Tallinn Estonia; 4 Motoneuron Functional Unit Hospital Universitari de Bellvitge Barcelona Spain

**Keywords:** amyotrophic lateral sclerosis, bulbar involvement, voice, diagnosis, machine learning

## Abstract

**Background:**

Bulbar involvement is a term used in amyotrophic lateral sclerosis (ALS) that refers to motor neuron impairment in the corticobulbar area of the brainstem, which produces a dysfunction of speech and swallowing. One of the earliest symptoms of bulbar involvement is voice deterioration characterized by grossly defective articulation; extremely slow, laborious speech; marked hypernasality; and severe harshness. Bulbar involvement requires well-timed and carefully coordinated interventions. Therefore, early detection is crucial to improving the quality of life and lengthening the life expectancy of patients with ALS who present with this dysfunction. Recent research efforts have focused on voice analysis to capture bulbar involvement.

**Objective:**

The main objective of this paper was (1) to design a methodology for diagnosing bulbar involvement efficiently through the acoustic parameters of uttered vowels in Spanish, and (2) to demonstrate that the performance of the automated diagnosis of bulbar involvement is superior to human diagnosis.

**Methods:**

The study focused on the extraction of features from the phonatory subsystem—jitter, shimmer, harmonics-to-noise ratio, and pitch—from the utterance of the five Spanish vowels. Then, we used various supervised classification algorithms, preceded by principal component analysis of the features obtained.

**Results:**

To date, support vector machines have performed better (accuracy 95.8%) than the models analyzed in the related work. We also show how the model can improve human diagnosis, which can often misdiagnose bulbar involvement.

**Conclusions:**

The results obtained are very encouraging and demonstrate the efficiency and applicability of the automated model presented in this paper. It may be an appropriate tool to help in the diagnosis of ALS by multidisciplinary clinical teams, in particular to improve the diagnosis of bulbar involvement.

## Introduction

### Background

Amyotrophic lateral sclerosis (ALS) is a neurodegenerative disease with an irregular and asymmetric progression, characterized by a progressive loss of both upper and lower motor neurons that leads to muscular atrophy, paralysis, and death, mainly from respiratory failure. The life expectancy of patients with ALS is between 3 and 5 years from the onset of symptoms. ALS produces muscular weakness and difficulties of mobility, communication, feeding, and breathing, making the patient heavily dependent on caregivers and relatives and generating significant social costs. Currently, there is no cure for ALS, but early detection can slow the disease progression [1].

The disease is referred to as spinal ALS when the first symptoms appear in the arms and legs (limb or spinal onset; 80% of cases) and bulbar ALS when it begins in cranial nerve nuclei (bulbar onset; 20% of cases). Patients with the latter form tend to have a shorter life span because of the critical nature of the bulbar muscle function that is responsible for speech and swallowing. However, 80% of all patients with ALS experience dysarthria, or unclear, difficult articulation of speech [2]. On average, speech remains adequate for approximately 18 months after the first bulbar symptoms appear [3]. These symptoms usually become noticeable at the beginning of the disease in bulbar ALS or in later stages of spinal ALS. Early identification of bulbar involvement in people with ALS is critical for improving diagnosis and prognosis and may be the key to effectively slowing progression of the disease. However, there are no standardized diagnostic procedures for assessing bulbar dysfunction in ALS.

Speech impairment may begin up to 3 years prior to diagnosis of ALS [3], and as ALS progresses over time there is significant deterioration in speech [4]. Individuals with ALS with severe dysarthria present specific speech production characteristics [5-7]. However, it is possible to detect early, often imperceptible, changes in speech and voice through objective measurements, as suggested in previous works [8-11]. The authors concluded that phonatory features may be well suited to early ALS detection.

### Related Work

Previous speech production studies have revealed significant differences in specific acoustic parameters in patients with ALS. Carpenter et al [7] studied the articulatory subsystem of individuals with ALS and found different involvement of articulators—that is, the tongue function was more involved than the jaw function. In a recent study, Shellikeri et al [5] found that the maximum speed of tongue movements and their duration were only significantly different at an advanced stage of bulbar ALS compared with the healthy control group. Connaghan et al [12] used a smartphone app to identify and track speech decline. Lee et al [6] obtained acoustic patterns for vowels in relation to the severity of the dysarthria in patients with ALS.

Other works have demonstrated the efficiency of features obtained from the phonatory subsystem for detecting early deterioration in ALS [8-11,13-15]. Studies have shown significant differences between jitter, shimmer, and the harmonics-to-noise ratio (HNR) in patients with ALS [8,10,11]. More specifically, Silbergleit et al [8] obtained these features from a steady portion of sustained vowels that provided information regarding changes in the vocal signal that are believed to reflect physiologic changes of the vocal folds. Alternative approaches used formant trajectories to classify the ALS condition [13], correlating formants with articulatory patterns [14], fractal jitter [15], Mel Frequency Cepstral Coefficients (MFCCs) [16], or combined acoustic and motion-related features [9] at the expense of introducing more invasive measurements to obtain data. Besides, the findings revealed significant differences in motion-related features only at an advanced stage of bulbar ALS.

Other related studies, such as one by Frid et al [17], used speech formants and their ratios to diagnose neurological disorders. Teixeira et al [18] and Mekyska et al [19] suggested jitter, shimmer, and HNR as good parameters to be used in intelligent diagnosis systems for dysphonia pathologies.

Garcia-Gancedo et al [20] demonstrated the feasibility of a novel digital platform for remote data collection of digital speech characteristics, among other parameters, from patients with ALS.

In the literature, classification models are widely used to test the performance of acoustic parameters in the analysis of pathological voices. Norel et al [21] identified acoustic speech features in naturalistic contexts and machine learning models developed for recognizing the presence and severity of ALS using a variety of frequency, spectral, and voice quality features. Wang et al [9] explored the classification of the ALS condition using the same features with support vector machine (SVM) and neuronal network (NN) classifiers. Rong et al [22] used SVMs with two feature selection techniques (decision tree and gradient boosting) to predict the intelligible speaking rate from speech acoustic and articulatory samples.

Suhas et al [16] implemented SVMs and deep neuronal networks (DNNs) for automatic classification by using MFCCs. An et al [23] used convolutional neuronal networks (CNNs) to compare the intelligible speech produced by patients with ALS to that of healthy individuals. Gutz et al [24] merged SVM and feature filtering techniques (SelectKBest). In addition, Vashkevich et al [25] used linear discriminant analysis (LDA) to verify the suitability of the sustain vowel phonation test for automatic detection of patients with ALS.

Among feature extraction techniques, principal component analysis (PCA) [26] shows good performance in a wide range of domains [27,28]. Although PCA is an unsupervised technique, it can efficiently complement a supervised classifier in order to achieve the objective of the system. In fact, any classifier can be used in conjunction with PCA because it does not make any kind of assumption about the subsequent classification model.

### Hypothesis

Based on previous works, our paper suggests that the acoustic parameters obtained through automated signal analysis from a steady portion of sustained vowels may be used efficiently as predictors for the early detection of bulbar involvement in patients with ALS. For that purpose, the main objectives (and contributions) of this research were (1) to design a methodology for diagnosing bulbar involvement efficiently through the acoustic parameters of uttered vowels in Spanish; and (2) to demonstrate that the performance of the automated diagnosis of bulbar involvement is superior to human diagnosis.

To fulfill these objectives, 45 Spanish patients with ALS and 18 control subjects took part in the study. They were recruited by a neurologist, and the five Spanish vowel segments were elicited from each participant. The study focused on the extraction of features from the phonatory subsystem—jitter, shimmer, HNR, and pitch—from the utterance of each Spanish vowel.

Once the features were obtained, we used various classification algorithms to perform predictions based on supervised classification. In addition to traditional SVMs [9,16,21,22,24], NNs [9,16,23], and LDA [25], we used logistic regression (LR), which is one of the most frequently used models for classification purposes [29,30]; random forest (RF) [31], which is an ensemble method in machine learning that involves the construction of multiple tree predictors that are classic predictive analytic algorithms [22]; and naïve Bayes (NaB), which is still a relevant topic [32] and is based on applying Bayes’ theorem.

Prior to feeding the models, PCA was applied to the features obtained due to the good performance observed of this technique in a wide range of domains.

## Methods

### Participants

The study was approved by the Research Ethics Committee for Biomedical Research Projects (CEIm) at the Bellvitge University Hospital in Barcelona, Spain. A total of 45 participants with ALS (26 males and 19 females) aged from 37 to 84 (mean 57.8, SD 11.8) years and 18 control subjects (9 males and 9 females) aged from 21 to 68 (mean 45.2, SD 12.2) years took part in this transversal study. All participants with ALS were diagnosed by a neurologist.

Bulbar involvement was diagnosed by following subjective clinical approaches [33], and the neurologist made the diagnosis of whether a patient with ALS had bulbar involvement. Of the 45 participants with ALS, 5 reported bulbar onset and 40 reported spinal onset, but at the time of the study 14 of them presented bulbar symptoms.

To summarize, of the 63 participants in the study, 14 were diagnosed with ALS with bulbar involvement (3 males and 11 females; aged from 38 to 84 years, mean 56.8 years, SD 12.3 years); 31 were diagnosed with ALS but did not display this dysfunction (23 males and 8 females, aged from 37 to 81 years, mean 58.3 years, SD 11.7 years); and 18 were control subjects (9 males and 9 females; aged from 21 to 68 years, mean 45.2 years, SD 12.2 years).

The severity of ALS and its bulbar presentation also varied among participants, as assessed by the ALS Functional Rating Scale-Revised (ALSFRS-R). The ALSFRS-R score (0-48) was obtained from 12 survey questions that assess the degree of functional impairment, with the score of each question ranging from 4 (least impaired) to 0 (most impaired). The scores of the 45 participants in this study ranged from 6 to 46 (mean 31.3, SD 8.6; 3 patients’ scores were reported as not available). Within the subgroups, the scores of patients diagnosed with bulbar involvement ranged from 6 to 46 (mean 23.1, SD 9.8), and the scores of participants with ALS who did not present this dysfunction ranged from 17 to 46 (mean 30.2, SD 8.0; 3 patients’ scores reported as not available).

The main clinical records of the participants with ALS are summarized in Multimedia Appendix 1.

### Vowel Recording

The Spanish phonological system includes five vowel segments—a, e, i, o, and u. These were obtained and analyzed from each patient with ALS and each control participant, all of whom were Spanish speakers.

Sustained samples of the Spanish vowels a, e, i, o, and u were elicited under medium vocal loudness conditions for 3-4 s. The recordings were made in a regular hospital room using a USB GXT 252 Emita Streaming Microphone (Trust International BV) connected to a laptop. The speech signals were recorded at a sampling rate of 44.100 Hz and 32-bit quantization using Audicity, an open-source application [34].

### Feature Extraction

Each individual phonation was cut out and anonymously labeled. The boundaries of the speech segments were determined with an oscillogram and a spectrogram using the Praat manual [35] and were audibly checked. The starting point of the boundaries was established as the onset of the periodic energy in the waveform observed in the oscillogram and checked by the apparition of the formants in the spectrogram. The end point was established as the end of the periodic oscillation when a marked decrease in amplitude in the periodic energy was observed. It was also identified by the disappearance of the waveform in the oscillogram and the formants in the spectrogram.

Acoustic analysis was done by taking into account the following features: jitter, shimmer, HNR, and pitch. Once the phonations of each participant had been segmented, the parameters were obtained from each vowel through the standard methods used in Praat [35]; they are explained in detail in this section and consist of a short-term spectral analysis and an autocorrelation method for periodicity detection.

Jitter and shimmer are acoustic characteristics of voice signals. Jitter is defined as the periodic variation from cycle to cycle of the fundamental period, and shimmer is defined as the fluctuation of the waveform amplitudes of consecutive cycles. Patients with lack of control of the vibration of the vocal folds tend to have higher values of jitter. A reduction of glottal resistance causes a variation in the magnitude of the glottal period correlated with breathiness and noise emission, causing an increase in shimmer [18].

To compute jitter parameters, some optional parameters in Praat were established. Period floor and period ceiling, defined as the minimum and maximum durations of the cycles of the waveform that were considered for the analysis, were set at 0.002 s and 0.025 s, respectively. The maximum period factor—the largest possible difference between two consecutive cycles—was set at 1.3. This means that if the period factor—the ratio of the duration of two consecutive cycles—was greater than 1.3, this pair of cycles was not considered in the computation of jitter.

The methods used to determine shimmer were almost identical to those used to determine jitter, the main difference being that jitter considers periods and shimmer takes into account the maximum peak amplitude of the signal.

Once the previous parameters had been established, jitter and shimmer were obtained by the formulas shown below [35].

Jitter(absolute) is the cycle-to-cycle variation of the fundamental period (ie, the average absolute difference between consecutive periods):

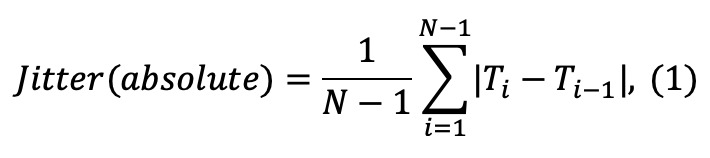



where *T_i_* is the duration of the *i*th cycle and *N* is the total number of cycles. If *T_i_* or *T_i_*_-1_ is outside the floor and ceiling periods, or if 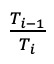 or 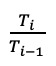 is greater than the maximum period factor, the term 
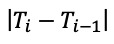
 is not counted in the sum, and *N* is lowered by 1 (if *N* ends up being less than 2, the result of the computation becomes “undefined”).

Jitter(relative) is the average absolute difference between consecutive periods divided by the average period. It is expressed as a percentage:

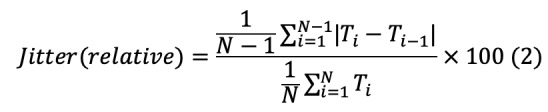



Jitter(rap) is defined as the relative average perturbation—the average absolute difference between a period and the average of this and its two neighbors, divided by the average period:

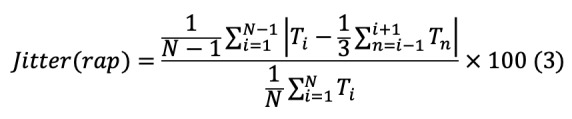



Jitter(ppq5) is the five-point period perturbation quotient, computed as the average absolute difference between a period and the average of this and its four closest neighbors, divided by the average period:

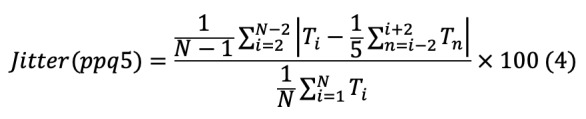



Shimmer(dB) is expressed as the variability of the peak-to-peak amplitude, defined as the difference between the maximum positive and the maximum negative amplitude of each period in decibels (ie, the average absolute base-10 logarithm of the difference between the amplitudes of consecutive periods, multiplied by 20:

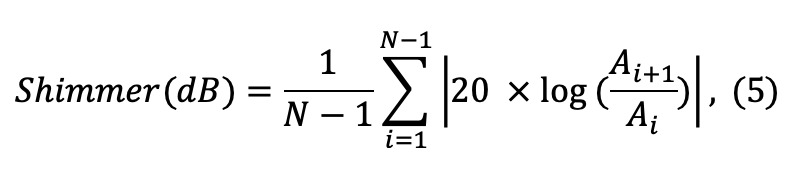



Where *A_i_* is the extracted peak-to-peak amplitude data and *N* is the number of extracted fundamental periods.

Shimmer(relative) is defined as the average absolute difference between the amplitudes of consecutive periods, divided by the average amplitude, expressed as a percentage:

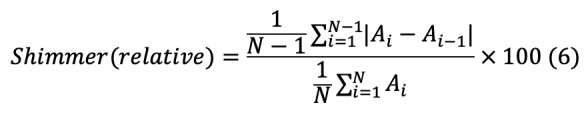



Shimmer(apq3) is the three-point amplitude perturbation quotient. This is the average absolute difference between the amplitude of a period and the average of the amplitudes of its neighbors, divided by the average amplitude:

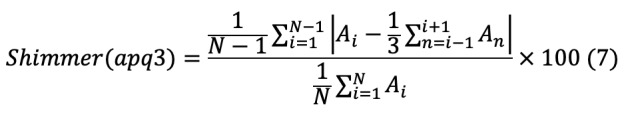



Shimmer(apq5) is defined as the five-point amplitude perturbation quotient, or the average absolute difference between the amplitude of a period and the average of the amplitudes of this and its four closest neighbors, divided by the average amplitude:

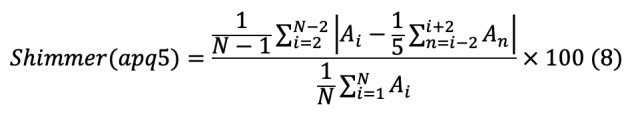



Shimmer(apq11) is expressed as the 11-point amplitude perturbation quotient, the average absolute difference between the amplitude of a period and the average of the amplitudes of this and its ten closest neighbors, divided by the average amplitude:

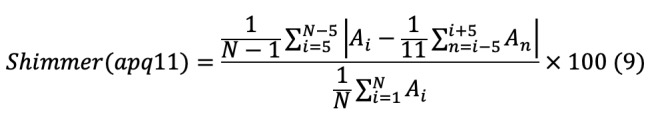



The HNR provides an indication of the overall periodicity of the voice signal by quantifying the ratio between the periodic (harmonics) and aperiodic (noise) components. The HNR was computed using Praat [35], based on the second maximum of normalized autocorrelation function detection, which is used in the following equation:

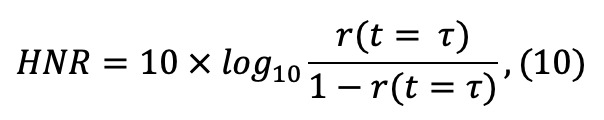



where *r*(*t*) is the normalized autocorrelation function, *r*(*t* = *τ*) is the second local maximum of the normalized autocorrelation and *τ* is the period of the signal.

The time step, defined as the measurement interval, was set at 0.01 s, the pitch floor at 60 Hz, the silence threshold at 0.1 (time steps that did not contain amplitudes above this threshold, relative to the global maximum amplitude, were considered silent), and the number of periods per window at 4.5, as suggested by Boersma and Weenink [35].

For the purpose of this study, the mean and standard deviation of the HNR were used.

To obtain the pitch, the autocorrelation method implemented in Praat [35] was used. The pitch floor for males and females was set at 60 Hz and 100 Hz, respectively, and the pitch ceiling for males and females was set at 300 Hz and 500 Hz, respectively. The time step was set, according to Praat [35], at 0.0075 s and 0.0125 s for females and males, respectively. Pitch above pitch ceiling and below pitch floor were not estimated. The mean and standard deviation of the pitch, as well as the minimum and maximum pitch, were features obtained from the pitch metric.

Textbox 1 shows the procedure, inspired by Praat [35], that was used to obtain the features explained above. The full code is freely available online [36].

**Algorithm for obtaining the features (jitter, shimmer, harmonics-to-noise ratio [HNR], and pitch) for acoustic analysis.**

1. Each individual phonation of each vowel was cut out and anonymously labeled to define the boundaries of the speech segments.
2. The values for the optional paramaters for analysis were set:
  Optional parameters to obtain jitter and shimmer parameters
    pitch floor: females 100 Hz and males 60 Hz
    pitch ceiling: females 500 Hz and males 300 Hz
    period floor: 0.002 s
    period ceiling: 0.025 s
    maximum period factor: 1.3
  Optional parameters to obtain HNR
    time step: 0.01 s
    pitch floor: 60 Hz
    silence threshold: 0.1
    number of periods per windows: 4.5
  Optional parameters to obtain pitch
    pitch floor: females 100 Hz and males 60 Hz
    pitch ceiling: females 500 Hz and males 300 Hz
    time step: females 0.0075 s and males 0.0125 s
3. Compute jitter and shimmer features—jitter(absolute), jitter(relative), jitter(rap), jitter(ppq5), shimmer(dB), shimmer(relative), shimmer(apq3), shimmer(apq5), shimmer(apq11)—using the configuration parameters established and then obtain the mean of each of these parameters for each vowel.
4. Compute HNR using the configuration parameters established and then obtain the mean (HNR[mean]) and standard deviation (HNR[SD]) values.
5. Compute pitch using the configuration parameters established and then obtain the mean (pitch[mean]), standard deviation (pitch[SD]), minimum (pitch[min]), and maximum (pitch[max]) values.
6. Obtain a data set with the 15 features computed.

### PCA

The PCA technique [37], a ranking feature extraction approach, was implemented in R [38] using the Stats package [38]. PCA was used to decompose the original data set into principal components (PCs) to obtain another data set whose data were linearly independent and therefore uncorrelated. It was performed by means of singular value decomposition (SVD) [39].

Prior to applying PCA, given that the mean age of control subjects was approximately 12 years younger than patients with ALS, we removed the age effects by using the data from the control subjects and applying the correction to all the participants as in the study by Norel et al [21]. We fitted the features extracted for healthy people and their age linearly. Then, the “normal aging” of each single feature of each participant was obtained by multiplying the age of the participants by the slope parameter obtained from the linear fit. Finally, the computed “normal aging” was removed from the features. Afterward, a standardized data set was obtained by subtracting the mean and centering the age-adjusted features at 0.

Then, by applying SVD to the standardized data set, a decomposition was obtained: 
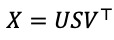
 , where *X* is the matrix of the standardized data set, *U* is a unitary matrix and *S* is the diagonal matrix of singular values *s_i_*. PCs are given by *US*, and *V* contains the directions in this space that capture the maximal variance of the features of the matrix *X*. The number of PCs obtained was the same as the original number of features, and the total variance of all of the PCs was equal to the total variance among all of the features. Therefore, all of the information contained in the original data was preserved.

From the PCA, a biplot chart was obtained for a visual appraisal of the data [40]. The biplot chart allowed us to visualize the data set structure, identify the data variability and clustering participants, and display the variances and correlations of the analyzed features. Then, the first eight PCs that explained almost 100% of the variance were selected to fit the classification models.

### Supervised Models

The participants in this study belonged to three different groups: the control group (n=18), patients with ALS with bulbar involvement (n=14), and patients with ALS without bulbar involvement (n=31). Each participant was properly labeled as control (C) if the subject was a control participant, ALS with bulbar (B) if the subject was a participant with ALS diagnosed with bulbar involvement, or ALS without bulbar (NB) if the subject was a participant with ALS without bulbar involvement. In addition, the ALS (A) label was added to every participant with ALS, with or without bulbar involvement.

Supervised models were built to obtain predictions by comparing the four labeled groups between them. Textbox 2 summarizes the procedure used to create proper classification models.

**Algorithm used to create the classification models.**

1. Building the data set: each participant was classified as C (control), B (amyotrophic lateral sclerosis [ALS] with bulbar involvement), or NB (ALS without bulbar involvement) according to the features extracted from the utterance of the five Spanish vowels and the categorical attributes of the bulbar involvement.
2. "Undefined" values were found in few participants when computing the shimmer(apq11) for a specific vowel. They were handled by computing the mean of this parameter for the other vowels uttered by the same participant.
3. The age effects were removed from the data set.
4. The values of the features obtained from the acoustic analysis were zero centered and scaled by using the following equation: (*x_i_* – 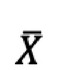 ) / *σ*, where *x_i_* is the feature vector, 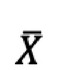 is the mean, and *σ* is the standard deviation. Scaling was performed to handle highly variable magnitudes of the features prior to computing primary component analysis (PCA).
5. The PCA was computed and a new data set was created with the first eight primary components (PCs).
6. A random seed was set to generate the same sequence of random numbers. They were used to divide the data set into chunks and randomly permute the data set. The random seed made the experiments reproducible and the classifier models comparable.
7. A 10-fold cross-validation technique was implemented and repeated for 10 trials. The data set was divided into ten contiguous chunks of approximately the same size. Then, 10 training-testing experiments were performed as follows: each chunk was held to test the classifier, and we performed training on the remaining chunks, applying upsampling with replacement by making the group distributions equal; the experiments were repeated for 10 trials, each trial starting with a random permutation of the data set.
8. Two different classification thresholds were established; 50% and 95% (more restrictive). The classification threshold is a value that dichotomizes the result of a quantitative test to a simple binary decision by treating the values above or equal to the threshold as positive and those below as negative.

Several supervised classification models were implemented in R [38] to measure the classification performance. The classification models were fitted with the first eight PCs that explained almost 100% of the data variability. Finally, 10-fold cross-validation was implemented in R using the caret package [41] to draw suitable conclusions. The upsampling technique with replacement was applied to the training data by making the group distributions equal to deal with the unbalanced data set, which could bias the classification models [42].

The first classifier employed was SVM, which is a powerful, kernel-based classification paradigm. SVM was implemented using the e1071 [43]. We used a C-support vector classification [44] and a linear kernel that was optimized through the tune function, assigning values of 0.0001, 0.0005, 0.001, 0.01, 0.1, and 1 to the C parameter, which controls the trade-off between a low training error and a low testing error. A C parameter value of 1 gave the best performance, and thus this was the SVM model chosen.

Next, a classical NN trained with the back propagation technique with an adaptive learning rate was implemented using the RSNNS package [45]. After running several trials to decide the NN architecture, a single hidden layer with three neurons was implemented because it showed the best performance. The activation function (transfer function) used was the hyperbolic tangent sigmoid function.

LDA was implemented using the MASS package [46]. It estimated the mean and variance in the training set and computed the covariance matrix to capture the covariance between the groups to make predictions by estimating the probability that the test set belonged to each of the groups.

LR was implemented by using the Gaussian generalized linear model applying the Stats package [38] for binomial distributions. A logit link function was used to model the probability of “success.” The purpose of the logit link was to take a linear combination of the covariate values and convert those values into a probability scale.

Standard NaB based on applying Bayes’ theorem was implemented using the e1071 package [43].

Finally, the RF classifier was implemented using the randomForest package [47] with a forest of 500 decision tree predictors. The optimal mtry—a parameter that indicated the number of PCs that were randomly distributed at each decision tree—was optimized for each classification problem by using the train function included in the caret package [41]. Each decision tree performed the classification independently and RF computed each tree predictor classification as one “vote.” The majority of the votes computed by all of the tree predictors decided the overall RF prediction.

The code of these implementations is freely available online [48].

### Performance Metrics

There are several metrics to evaluate classification algorithms [49]. The analysis of such metrics and their significance must be interpreted correctly to evaluate these algorithms.

There are four possible results in the classification task. If the sample is positive and it is classified as positive, it is counted as a true positive (TP), and when it is classified as negative, it is considered a false negative (FN). If the sample is negative and it is classified as negative or positive, it is considered a true negative (TN) or false positive (FP), respectively. Based on that, three performance metrics, presented below, were used to evaluate the performance of the classification models.

- Accuracy: ratio between the correctly classified samples.

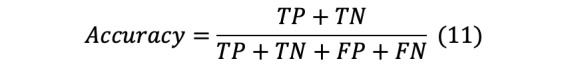

- Sensitivity: proportion of correctly classified positive samples compared with the total number of positive samples.

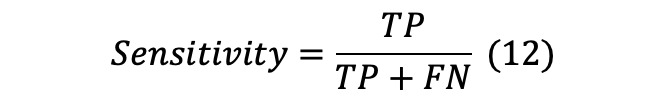

- Specificity: proportion of correctly classified negative samples compared with the total number of negative samples.

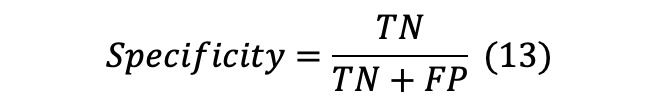

Finally, paired Bonferroni-corrected Student *t* tests [50] were implemented to evaluate the statistical significance of the metrics results. To reject the null hypothesis, which entails considering that there is no difference in the performance of the classifiers, a significance level of α=.05 was established for all tests. The *P* values obtained by performing the tests with values below α=.05 rejected the null hypothesis.

## Results

First, the distributions of the features obtained were examined. Then, the PCA was performed and the supervised models studied were evaluated.

### Data Exploration

A total of 15 features were obtained in this study. These features were jitter(absolute), jitter(relative), jitter(rap), jitter(ppq5), shimmer(relative), shimmer(dB), shimmer(apq3), shimmer(apq5), shimmer(apq11), pitch(mean), pitch(SD), pitch(min), pitch(max), HNR(mean), and HNR(SD).

**Figure 1:**
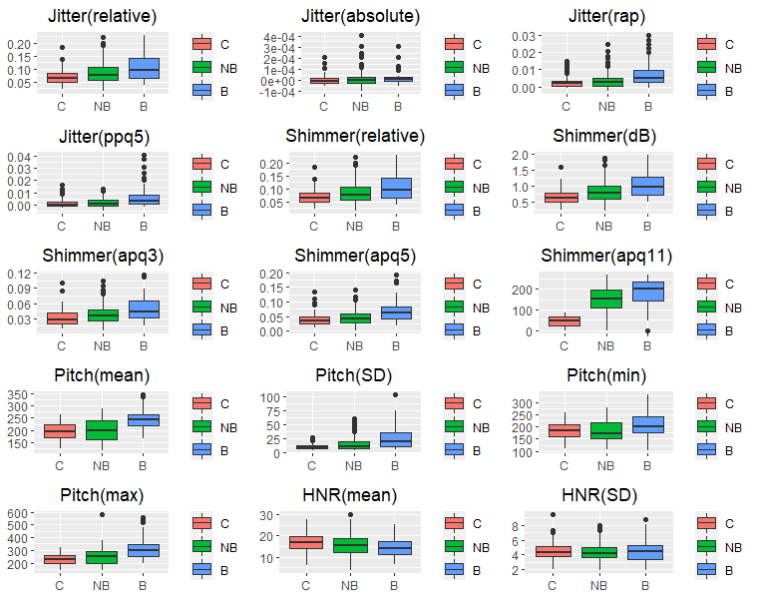
Box plots of features by group. B: patients with amyotrophic lateral sclerosis (ALS) with bulbar involvement; C: control group; HNR: harmonics-to-noise ratio; NB: patients with ALS without bulbar involvement.

Figure 1 shows the box plot of the features obtained from the control (C) group, patients with ALS with bulbar involvement (B), and patients with ALS without bulbar involvement (NB). The means in the B group were higher than those in the C and NB groups. The means in the NB group were located in the middle of the means of the C and B groups. On the contrary, the B group obtained the lowest values for the mean HNR(mean) and HNR(SD). Differences in the standard deviation between the three groups were also observed. In general, features obtained from the B group presented the highest standard deviations.

### PCA

**Figure 2:**
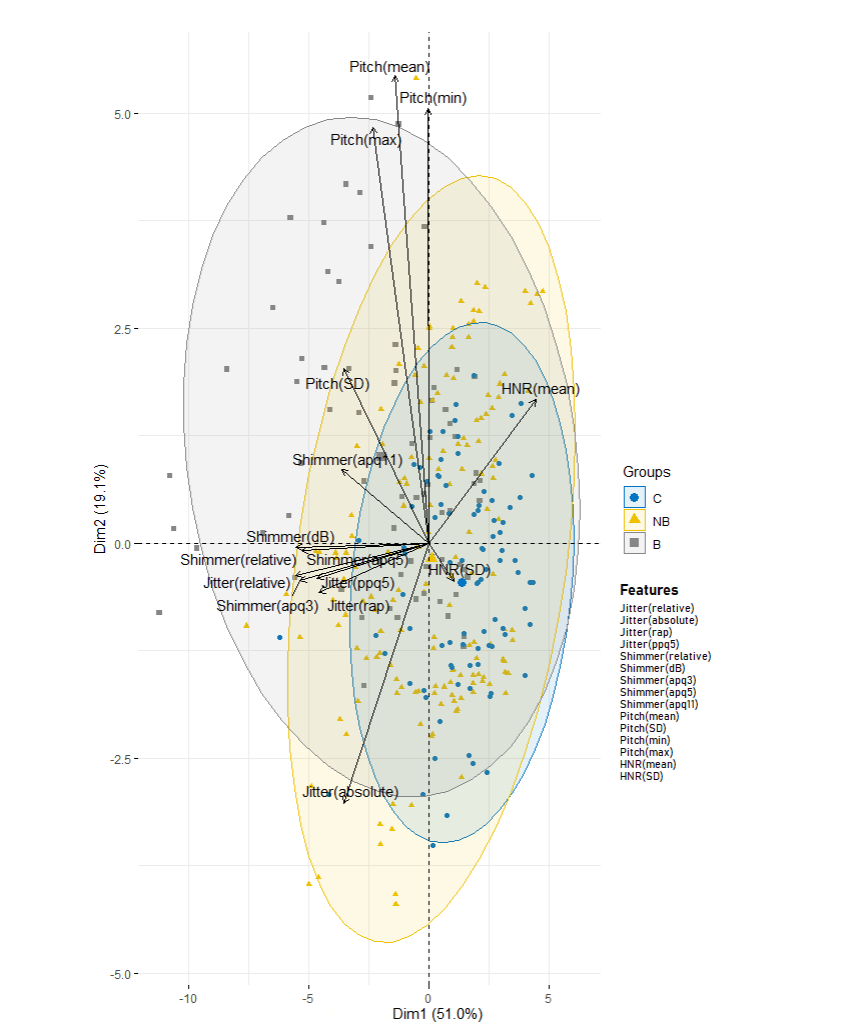
Principal component analysis biplot chart representing the variance of the first (Dim1) and second (Dim2) principal components in the control group (C), patients with amyotrophic lateral sclerosis (ALS) without bulbar involvement (NB), and patients with ALS with bulbar involvement (B). HNR: harmonics-to-noise ratio.

PCA was performed using the data set that contained the 15 features extracted from all of the participants. Figure 2 shows the associated PCA biplot chart. The two axes represent the first (Dim1) and second (Dim2) PCs. The biplot uses the diagonalization method to give a graphical display of its dimensional approximation [51,52]. The interpretation of the biplot involves observing the lengths and directions of the vectors of the features, the data variability, and the clusterization of the participants.

It can be observed that a considerable proportion of variance (70.1%) of the shimmer, jitter, pitch, and HNR was explained. The relative angle between any two vector features represents their pairwise correlation. The closer the vectors are to each other (<90°), the higher their correlation. When vectors are perpendicular (angles of 90° or 270°), the variables have a small or null correlation. Angles approaching 0° or 180° (collinear vectors) indicate a correlation of 1 or –1, respectively. Thus, in this case, shimmer and jitter show a strong positive correlation. Another important observation reflected in Figure 2 is the spatial proximity of the groups in relation both to each other and to the set of features. The projection of the B group onto the vector for shimmer and jitter falls to the left of the vector features. This means that subjects labelled as the B group had higher average values for those features than the average values of the other groups. Conversely, the projection of the C group onto those variables falls on the opposite side. In addition, the C and B groups are more distant from each other when projected onto shimmer and jitter. This indicates that shimmer and jitter features are the most important features for the classification of participants in the B and C groups.

The projection of subjects in the NB group requires special attention. Although the projection of these subjects has a spatial proximity with respect to the C group, their variability is higher, overflowing the gray circle corresponding to the B group.

This indicates that some features, especially shimmer and jitter, of some subjects in the NB group have similar projections to the features of the B group.

**Figure 3:**
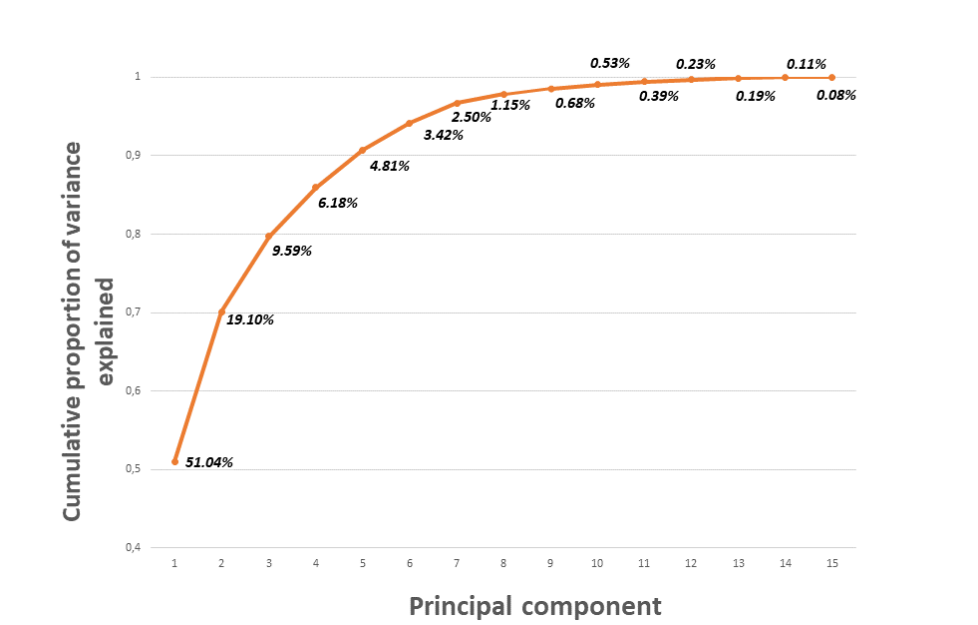
Cumulative percentage of the explained variance using principal component analysis.

To fit the models, as explained in detail in the next section, the first eight PCs were selected in order to reduce the dimensionality but preserve almost 100% of the variability as shown in Figure 3.

### Supervised Model Evaluation

The first eight PCs were selected. Then, each classification model was applied to these PCs. Consequently, better results were obtained than when applying the classification models alone. The results of the classification methods alone are not shown because of their limited contribution to the analysis.

Tables 1 and 2 show the classification performance (accuracy, sensitivity, and specificity metrics) of the supervised models tested for the four cases with the classification threshold set at 50% and 95%, respectively.

**Table 1:** Classification performance of the supervised models with the classification threshold set at 50%.

		Classification performance (%)			
Model and metrics		C^a^ vs B^b^	C vs NB^c^	B vs NB	C vs ALS^d^
**Random forest**					
	Accuracy	93.6	91.1	75.5	90.3
	Sensitivity	91.1	92.1	55.7	92.1
	Specificity	95.5	89.6	88.4	85.7
**Naïve Bayes**					
	Accuracy	91.0	87.9	75.4	90.3
	Sensitivity	89.2	86.7	62.7	92.1
	Specificity	93.2	90.0	81.2	85.7
**Logistic regression**					
	Accuracy	93.8	91.4	70.1	91.1
	Sensitivity	92.5	89.1	62.2	89.6
	Specificity	94.8	95.6	73.5	93.3
**Linear discriminant analysis**					
	Accuracy	94.3	91.6	71.2	91.6
	Sensitivity	95.6	87.4	61.8	88.3
	Specificity	90.0	98.8	75.4	87.8
**Neuronal network**					
	Accuracy	94.8	92.5	70.4	92.2
	Sensitivity	91.7	90.3	60.0	90.8
	Specificity	97.2	96.4	75.2	95.6
**Support vector machine**					
	Accuracy	95.8	91.5	69.9	91.6
	Sensitivity	91.4	88.4	59.4	88.9
	Specificity	99.3	97.0	74.6	98.2

^a^C: control group.

^b^B: patients with amyotrophic lateral sclerosis (ALS) with bulbar involvement.

^c^NB: patients with ALS without bulbar involvement.

^d^ALS: all patients with ALS.

**Table 2:** Classification performance of the supervised models with the classification threshold set at 95%.

		Classification performance (%)			
Model and metrics		C^a^ vs B^b^	C vs NB^c^	B vs NB	C vs ALS^d^
**Random forest**					
	Accuracy	58.3	56.1	68.8	75.1
	Sensitivity	4.8	30.4	0.0	65.6
	Specificity	100.0	100.0	100.0	98.8
**Naïve Bayes**					
	Accuracy	82.3	68.8	72.8	75.1
	Sensitivity	64.7	54.6	15.8	65.6
	Specificity	96.1	93.3	98.6	98.8
**Logistic regression**					
	Accuracy	92.8	77.7	74.1	76.0
	Sensitivity	84.8	65.1	16.7	66.4
	Specificity	99.0	99.6	100.0	100.0
**Linear discriminant analysis**					
	Accuracy	88.1	70.6	71.7	71.1
	Sensitivity	72.7	53.5	0.9	59.5
	Specificity	100.0	100.0	100.0	100.0
**Neuronal network**					
	Accuracy	92.6	84.8	73.1	86.8
	Sensitivity	83.2	76.1	20.5	81.6
	Specificity	100.0	100.0	96.8	99.8
**Support vector machine**					
	Accuracy	86.3	71.1	70.7	71.1
	Sensitivity	68.8	54.3	6.1	59.4
	Specificity	100.0	100.0	100.0	100.0

^a^C: control group.

^b^B: patients with amyotrophic lateral sclerosis (ALS) with bulbar involvement.

^c^NB: patients with ALS without bulbar involvement.

^d^ALS: all patients with ALS.

In the case of the C group versus the B group, with the classification threshold set at 50%, the results indicated that all classifiers had a good classification performance. SVM obtained the best accuracy (95.8%). The tests of significance, which are reported in Multimedia Appendix 2, revealed statistically significant differences between SVM and the other models, with the exception of LDA, which obtained an accuracy (94.3%) that closely approximated that of the SVM model. NN also showed really good results (accuracy 94.8%).

Similar behavior was obtained in the C group versus the NB group and the C group versus all patients with ALS. In these cases, NN was the best model (92.5% for C vs NB and 92.2% for C versus ALS). Meanwhile, generally poor performance was obtained in the B group versus the NB group compared with the other cases. Although RF showed the best accuracy (75.5%), the performance of specificity and especially sensitivity dropped dramatically in comparison with the previous cases. In general, the model performance dropped with a 95% threshold. In the C group versus the B group, the accuracy of the classification models (Table 2) was worse than when the classification threshold was set at 50%. LR shows the best accuracy (92.8%). LDA, SVM, and NaB obtained accuracies of 88.1%, 86.3%, and 82.3%, respectively. RF did not seem to be a good model for this threshold, with an accuracy of 58.3%.

Lower results were obtained in the C group versus the NB group and the C group versus the group with ALS. NN showed the best performance, with accuracies of 84.8% and 86.8%, respectively.

With the 95% threshold, the performance of sensitivity dropped in all cases, especially for the B group versus the NB group, where LR obtained the best performance with an accuracy of 74.1% but a sensitivity of 16.7%.

## Discussion

### Principal Findings

This study was guided by 2 objectives: (1) to design a methodology for diagnosing bulbar involvement efficiently through the acoustic parameters of uttered vowels in Spanish, and (2) to demonstrate the superior performance of automated diagnosis of bulbar involvement compared with human diagnosis. This was based on the accurate acoustic analysis of the five Spanish vowel segments, which were elicited from all participants. A total of 15 acoustic features were extracted: jitter(absolute), jitter(relative), jitter(rap), jitter(ppq5), shimmer(relative), shimmer(dB), shimmer(apq3), shimmer(apq5), shimmer(apq11), pitch(mean), pitch(SD), pitch(min), pitch(max), HNR(mean), and HNR(SD). Then, the PCs of these features were obtained to fit the most common supervised classification models in clinical diagnosis: SVM, NN, LDA, LR, NaB, and RF. Finally, the performance of the models was compared.

The study demonstrated the feasibility of automatic detection of bulbar involvement in patients with ALS through acoustic features obtained from vowel utterance. It also confirms that speech impairment is one of the most important aspects for diagnosing bulbar involvement, as was suggested by Pattee et al [33]. Furthermore, bulbar involvement can be detected using automatic tools before it becomes perceptible to human hearing.

Voice features extracted from the B group compared with those features extracted from the C group showed the best performance of the classification model for determining bulbar involvement in patients with ALS.

Accuracy for the C group versus the B group revealed values of 95.8% for SVM with the classification threshold established at 50%. However, on increasing the threshold to 95%, the accuracy values for SVM dropped (86.3%) and LR showed the best performance (accuracy 92.8%). NN also showed a good accuracy at 92.6%. This implies that NN and LR are more robust for finding accuracy.

For that case, the results obtained reinforce the idea that it is possible to diagnose bulbar involvement in patients with ALS using supervised models and objective measures. The SVM and LR models provided the best performance for the 50% and 95% thresholds, respectively.

Great uncertainty was found in the analysis regarding bulbar involvement in the NB group. The accuracy values of the C group versus the NB group and the C group versus the group with ALS with the classification threshold at 50% were 92.5% and 92.2%, respectively, for NN. That reveals that the features extracted from the NB group differed significantly from those of the C group. Lower performance should be expected because participants labeled as the C group and NB group should have similar voice performance. This may indicate that some of the participants in the NB group probably had bulbar involvement but were not correctly diagnosed because the perturbance in their voices could not be appreciated by the human ear. Alternatively, it could be simply that a classification threshold of 50% was too optimistic. With a 95% classification threshold, lower results were obtained in the C group versus the NB group and in the C group versus patients with ALS. NN showed the best performance with accuracies of 84.8% and 86.8%, respectively, for the two cases.

The performance between the B group and C group showed better results than between the NB group and C group. Despite this, the unexpectedly high performance of the models for the C group versus the NB group still suggests that some participants in the NB group could have had bulbar involvement. Changing the classification threshold to 95% worsened the results, especially for sensitivity, although this still remained significant.

The case of the B group versus the NB group revealed that the classification models did not distinguish B group and NB group participants as well as they did with the other groups. The accuracy with the 50% threshold showed the highest performance for RF (75.5%), but the models showed difficulties in identifying positive cases. That may be due to the small difference in the variation of the data among participants in the B and NB groups. The same occurred for the 95% threshold: LR obtained the highest accuracy (74.1%) but a sensitivity of only 16.7%. These values remain far from those in the case of the C group versus the B group. These results also reinforce the idea that participants in the NB group were misdiagnosed.

The good model performance obtained in comparing the C and NB groups supports these findings and underscores the importance of using objective measures for assessing bulbar involvement. This corroborated the results obtained in the data exploration and PCA, which were presented in the Results section.

The projection of the NB group in the PCA biplot chart requires special attention. Although the projection of these subjects has a spatial proximity with regard to the C group, their variability is higher, overflowing the circle corresponding to the B group. This indicates that some features, especially shimmer and jitter, of some patients in the NB group have similar projections to those in the B group. This may reveal that these patients in the NB group could have bulbar involvement but were not yet correctly diagnosed because the perturbance in their voices could still not be appreciated by human hearing.

Figure 1 also indicates that the means of the features of the patients in the NB group were between the means of the features of the C and B groups, thus corroborating these assumptions.

### Limitations

This study has some limitations. First, using machine learning on small sample sizes makes it difficult to fully evaluate the significance of the findings. The sample size of this study was heavily influenced by the fact that ALS is a rare disease. At the time of the study, 14 of the patients with ALS presented bulbar symptoms. The relatively small size of this group was because ALS is a very heterogeneous disease and not all patients with ALS present the same symptomatology. Additionally, the control subjects were approximately 12 years younger than the patients with ALS. Vocal quality changes with age, and comparing younger control subjects’ vocalic sounds with those of older participants with ALS might introduce additional variations. Although upsampling techniques were used in this study to correct the bias and age adjustments have been applied to correct the vocal quality changes due to the age difference, it would be necessary in future studies to increase the number of participants, especially of patients with ALS with bulbar involvement and control participants of older ages, to draw definitive conclusions.

Second, the variability inherent in establishing the boundaries of the speech segments on spectrograms manually makes replicability challenging. Speakers will differ in their production, and even the same speaker in the same context will not produce two completely identical utterances. In this study, the recorded speech was processed manually in the uniform approach detailed in the Methods section. Automatic instruments have been developed, but unfortunately these methods are not yet accurate enough and require manual correction.

### Comparison with Prior Work

The PCA biplot charts indicated that shimmer and jitter were the most important features for group separation in the 2-PC model for ALS classification; however, they also revealed pitch and HNR parameters as good variables for this purpose. These results are consistent with those of Vashkevich et al [25], who demonstrated significant differences in jitter and shimmer in patients with ALS. They are also consistent with Mekyska et al [19] and Teixeira et al [18], who mentioned pitch, jitter, shimmer, and HNR values as the most popular features describing pathological voices. Finally, Silbergleit et al [8] suggested that the shimmer, jitter, and HNR parameters are sensitive indicators of early laryngeal deterioration in ALS.

Concerning the classification models, Norel et al [21] recently implemented SVM classifiers to recognize the presence of speech impairment in patients with ALS. They identified acoustic speech features in naturalistic contexts, achieving 79% accuracy (sensitivity 78%, specificity 76%) for classification of males and 83% accuracy (sensitivity 86%, specificity 78%) for classification of females. The data used did not originate from a clinical trial or contrived study nor was it collected under laboratory conditions. Wang et al [9] implemented SVM and NN using acoustic features and adding articulatory motion information (from tongue and lips). When only acoustic data were used to fit the SVM, the overall accuracy was slightly higher than the level of chance (50%). Adding articulatory motion information further increased the accuracy to 80.9%. The results using NN were more promising, with accuracies of 91.7% being obtained using only acoustic features and increasing to 96.5% with the addition of both lip and tongue data. Adding motion measures increased the classifier accuracy significantly at the expense of including more invasive measurements to obtain the data. We investigated the means of optimizing accuracy in detecting ALS bulbar involvement by only analyzing the voices of patients. An et al [23] implemented CNNs to classify the intelligible speech produced by patients with ALS and healthy individuals. The experimental results indicated a sensitivity of 76.9% and a specificity of 92.3%. Vashkevich et al [25] performed LDA with an accuracy of 90.7% and Suhas et al [16] used DNNs based on MFCCs with an accuracy of 92.2% for automatic detection of patients with ALS.

Starting with the most widely used features suggested in the literature, the classification models used in this paper to detect bulbar involvement automatically (C group versus B group) performed better than the ones used by other authors, specifically the ones obtained using NN (Wang et al [9]) and DNNs based on MCCFs (Suhas et al [16]). We obtained the best-ever performance metrics. This suggests that decomposing the original data set of features into PCs to obtain another data set whose data (ie, PCs) were linearly independent and therefore uncorrelated improves the performance of the models.

### Conclusions

This paper suggests that machine learning may be an appropriate tool to help in the diagnosis of ALS by multidisciplinary clinical teams. In particular, it could help in the diagnosis of bulbar involvement. This work demonstrates that an accurate analysis of the features extracted from an acoustic analysis of the vowels elicited from patients with ALS may be used for early detection of bulbar involvement. This could be done automatically using supervised classification models. Better performance was achieved by applying PCA previously to the obtained features. It is important to note that when classifying participants with ALS with bulbar involvement and control subjects, the SVM with a 50% classification threshold exceeded the performance obtained by other authors, specifically Wang et al [9] and Suhas et al [16].

Furthermore, bulbar involvement can be detected using automatic tools before it becomes perceptible to human hearing. The results point to the importance of obtaining objective measures to allow an early and more accurate diagnosis, given that humans may often misdiagnose this deficiency. This directly addresses a recent statement released by the Northeast ALS Consortium’s bulbar subcommittee regarding the need for objective-based approaches [53].

### Future Work

Future work is directed toward the identification of incorrectly undiagnosed bulbar-involvement in patients with ALS. A time-frequency representation will be used to detect possible deviations in the voice performance of patients in the time-frequency domain. The voice distributions of patients with ALS diagnosed with bulbar involvement and patients with ALS without that diagnosis will be compared in order to detect pattern differences between these two groups. That could provide indications to distinguish undiagnosed participants with ALS who could be misdiagnosed. Also, an improvement in the voice database by increasing the sample size is envisaged.

## Appendix

Multimedia Appendix 1 Summary of the clinical records of participants with amyotrophic lateral sclerosis.

Multimedia Appendix 2 Paired t test with Bonferroni correction.

## Abbreviations

ALS: amyotrophic lateral sclerosis
ALSFRS-R: ALS Functional Rating Scale-Revised
CNN: convolutional neuronal network
DNN: deep neuronal network
FN: false negative
FP: false positive
HNR: harmonics-to-noise ratio
LDA: linear discriminant analysis
LR: logistic regression
MFCC: Mel Frequency Cepstral Coefficient
NaB: naïve Bayes
NN: neuronal network
PC: principal component
PCA: principal component analysis
RF: random forest
SVD: singular value decomposition
SVM: support vector machine
TN: true negative
TP: true positive
