# Supplementary material for: Detection of Bulbar Involvement in Patients With Amyotrophic Lateral Sclerosis by Machine Learning Voice Analysis: Diagnostic Decision Support Development Study
Source: JMIR Med Inform. 2021 Mar 10;9(3):e21331. doi: 10.2196/21331 (PMC7991994; doi:10.2196/21331)
Supplement: Multimedia Appendix 1 [file medinform_v9i3e21331_app1.pdf]

Multimedia Appendix: ALS participants. Clinical Records.

This is a Multimedia Appendix to a full manuscript published in the J Med Internet Res. For full copyright and citation information see http://dx.doi.org/10.2196/jmir.21331

TABLE I: ALS Participants Clinical Records.

**ID**	**Age** *(years)*	**Sex**	**Onset Type**	**ALSFR-R** *(Total Score)*	**Bulbar Involvement**	**Type** *(Bulbar Involvement)*
HUB001	53	F	SPINAL	29	YES	Dysphagia/Dysarthria
HUB002	52	F	BULBAR	27	YES	Dysphagia
HUB003	71	M	SPINAL	39	NO	No Symptoms
HUB004	52	M	SPINAL	43	NO	No Symptoms
HUB005	49	M	SPINAL	NA	NO	No Symptoms
HUB006	70	F	BULBAR	23	YES	Dysphagia/Dysarthria
HUB007	70	M	SPINAL	27	NO	No Symptoms
HUB008	67	F	SPINAL	33	YES	Dyspnoea
HUB009	71	F	SPINAL	32	YES	Dysphagia
HUB010	72	M	SPINAL	30	NO	No Symptoms
HUB011	43	F	SPINAL	21	YES	Dysphagia
HUB012	70	F	SPINAL	28	YES	Dysphagia
HUB013	41	M	SPINAL	34	NO	No Symptoms
HUB014	70	M	SPINAL	46	NO	No Symptoms
HUB015	58	F	BULBAR	46	YES	Dysarthria
HUB016	55	M	SPINAL	26	NO	No Symptoms
HUB017	48	F	SPINAL	36	NO	No Symptoms
HUB018	58	M	SPINAL	28	YES	NA
HUB019	63	M	SPINAL	22	NO	No Symptoms
HUB020	50	M	SPINAL	39	NO	No Symptoms
HUB021	84	F	BULBAR	30	YES	NA
HUB022	52	M	SPINAL	33	NO	No Symptoms
HUB023	56	M	SPINAL	27	NO	No Symptoms
HUB024	65	M	SPINAL	24	NO	No Symptoms
HUB025	68	F	SPINAL	21	NO	No Symptoms
HUB026	66	F	SPINAL	41	NO	No Symptoms
HUB027	81	M	SPINAL	28	NO	No Symptoms
HUB028	44	F	SPINAL	19	NO	No Symptoms
HUB029	70	F	SPINAL	17	NO	No Symptoms
HUB030	72	F	SPINAL	38	NO	No Symptoms
HUB031	60	M	BULBAR	46	YES	NA
HUB032	48	M	SPINAL	31	NO	No Symptoms
HUB033	59	F	SPINAL	33	YES	NA
HUB034	37	F	SPINAL	37	NO	No Symptoms
HUB035	76	F	SPINAL	30	NO	No Symptoms
HUB036	48	F	SPINAL	29	YES	Dysphagia
HUB037	69	M	SPINAL	37	NO	No Symptoms
HUB038	81	M	SPINAL	36	NO	No Symptoms
HUB039	68	M	SPINAL	NA	NO	No Symptoms
HUB040	55	M	SPINAL	24	NO	No Symptoms
HUB041	38	M	SPINAL	6	YES	NA
HUB042	56	M	SPINAL	35	NO	No Symptoms
HUB043	67	M	SPINAL	NA	NO	No Symptoms
HUB044	63	M	SPINAL	42	NO	No Symptoms
HUB045	48	M	SPINAL	45	NO	No Symptoms
