# Supplementary material for: Detection of Bulbar Involvement in Patients With Amyotrophic Lateral Sclerosis by Machine Learning Voice Analysis: Diagnostic Decision Support Development Study
Source: JMIR Med Inform. 2021 Mar 10;9(3):e21331. doi: 10.2196/21331 (PMC7991994; doi:10.2196/21331)
Supplement: Multimedia Appendix 2 [file medinform_v9i3e21331_app2.pdf]

Multimedia Appendix: Paired t-test with Bonferroni correction.

This is a Multimedia Appendix to a full manuscript published in the J Med Internet Res. For full copyright and citation information see http://dx.doi.org/10.2196/jmir.21331

	Supervised Classification Models				
***P* values**	LDA	LR	NaB	NN	RF
LR	$<$ .001	-	-	-	-
NaB	$<$ .001	$<$ .001	-	-	-
NN	.04	.03	$<$ .001	-	-
RF	$<$ .001	.69	.01	.01	-
SVM	.37	.04	$<$ .001	$<$ .001	.003

TABLE I: Paired t-test with Bonferroni correction for C vs. B with a classification threshold of 50%.

	Supervised Classification Models				
***P* values**	LDA	LR	NaB	NN	RF
LR	.57	-	-	-	-
NaB	$<$ .001	$<$ .001	-	-	-
NN	.02	.01	$<$ .001	-	-
RF	.44	.67	$<$ .001	.008	-
SVM	.84	.58	$<$ .001	.009	.50

TABLE II: Paired t-test with Bonferroni correction for C vs. NB with a classification threshold of 50%.

	Supervised Classification Models				
***P* values**	LDA	LR	NaB	NN	RF
LR	.003	-	-	-	-
NaB	.005	$<$ .001	-	-	-
NN	.44	.61	.00172	-	-
RF	$<$ .001	$<$ .001	.89	$<$ .001	-
SVM	.01701	.82	$<$ .001	.54	$<$ .001

TABLE III: Paired t-test with Bonferroni correction for B vs. NB with a classification threshold of 50%.

	Supervised Classification Models				
***P* values**	LDA	LR	NaB	NN	RF
LR	.76	-	-	-	-
NaB	$<$ .001	$<$ .001	-	-	-
NN	.08	.04	$<$ .001	-	-
RF	.002	.004	$<$ .001	.004	-
SVM	.86	.83	$<$ .001	.02	.002

TABLE IV: Paired t-test with Bonferroni correction for C vs. ALS with a classification threshold of 50%.

P values	Supervised Classification Models				
	LDA	LR	NaB	NN	RF
LR	$< .001$	-	-	-	-
NaB	$< .001$	$< .001$	-	-	-
NN	$< .001$	.69	$< .001$	-	-
RF	$< .001$	$< .001$	$< .001$	$< .001$	-
SVM	.003	$< .001$	.09	$< .001$	$< .001$

TABLE V: Paired t-test with Bonferroni correction for C vs. B with a classification threshold of 95%.

P values	Supervised Classification Models				
	LDA	LR	NaB	NN	RF
LR	$< .001$	-	-	-	-
NaB	.008	$< .001$	-	-	-
NN	$< .001$	$< .001$	$< .001$	-	-
RF	.002	$< .001$	$< .001$	$< .001$	-
SVM	.83	$< .001$	.014	$< .001$	.001

TABLE VI: Paired t-test with Bonferroni correction for C vs. NB with a classification threshold of 95%.

P values	Supervised Classification Models				
	LDA	LR	NaB	NN	RF
LR	.002	-	-	-	-
NaB	$< .001$	.03	-	-	-
NN	.07	.68	.09	-	-
RF	$< .001$	$< .001$	$< .001$	$< .001$	-
SVM	.005	$< .001$	$< .001$	$< .001$	.06

TABLE VII: Paired t-test with Bonferroni correction for B vs. NB with a classification threshold of 95%.

P values	Supervised Classification Models				
	LDA	LR	NaB	NN	RF
LR	$< .001$	-	-	-	-
NaB	.34	.04	-	-	-
NN	$< .001$	$< .001$	$< .001$	-	-
RF	.71	$< .001$	.23	$< .001$	-
SVM	.37	$< .001$	.48	$< .001$	.42

TABLE VIII: Paired t-test with Bonferroni correction for C vs. ALS with a classification threshold of 95%.
